# Is measurement of the gingival biotype reliable? Agreement among different assessment methods

**DOI:** 10.4317/medoral.23280

**Published:** 2019-12-24

**Authors:** Laura Aguilar-Duran, Javier Mir-Mari, Rui Figueiredo, Eduard Valmaseda-Castellón

**Affiliations:** 1 DDS, MS. Master of Oral Surgery and Implantology. Faculty of Medicine and Health Sciences, University of Barcelona; 2 DDS, MS. Master of Oral Surgery and Implantology. Professor of the Master in Oral Surgery and Implantology. Faculty of Medicine and Health Sciences, University of Barcelona; 3 DDS, MS, PhD. Master of Oral Surgery and Implantology. Associated Professor of Oral Surgery. Professor of the Master in Oral Surgery and Implantology. Faculty of Medicine and Health Sciences, University of Barcelona. Researcher at the IDIBELL Institute; 4 DDS, MS, PhD. Master of Oral Surgery and Implantology. Professor of Oral Surgery. Director of the Master in Oral Surgery and Implantology. Faculty of Medicine and Health Sciences, University of Barcelona. Researcher at the IDIBELL institute. Barcelona, Spain

## Abstract

**Background:**

To determine agreement among the most commonly used methods for assessing the gingival biotype.

**Material and Methods:**

An electronic survey was sent to a sample of dentists practicing in Spain. The questionnaire was based on the evaluation of 5 cases involving different gingival biotype assessment methods. Dentists were required to classify the cases as having a “thin”, “thick” or “not able to classify” biotype. Each case was assessed using a frontal intraoral photo of the anterior teeth; an enlarged photo of the buccal aspect of the tooth with a periodontal probe inserted inside the sulcus; and the real thickness measured in mm with a calibrated needle. Agreement among the classifications was assessed using Cohen’s kappa coefficient.

**Results:**

A total of 104 surveys were analyzed. The most commonly used assessment method was visual evaluation of the morphology of the gingiva and the teeth (62.5%). Concordance among the three different methods was weak (kappa = 0.278). Agreement among the classification methods was greater in extreme cases (thinner and thicker gingival thickness).

**Conclusions:**

The most commonly used methods for assessing gingival biotype are not reliable. The three tested methods show poor to weak agreement, which leads to non-reliable estimation of the gingival biotype.

** Key words:**Gingival biotype, measurement methods, visual assessment, gingival thickness.

## Introduction

Gingival biotype is considered to be an important variable, since it influences the treatment outcomes in periodontal and implant treatments (1-3). The first classification to be described was based on the gingival anatomy and the shape of the dental crown, and the biotype was classified as either “thin-scalloped type” or “thick-flat type” (4-5). A broader classification was subsequently developed based on the gingival thickness, gingival morphotype, tooth dimensions, keratinized tissue and bone morphotype: “Thin-scalloped biotype” for subjects with slender triangular shaped crown, a highly scalloped gingival margin, a narrow zone of keratinized tissue, interproximal contacts close to the incisal edge and a relative thin alveolar bone, “Thick-flat biotype” for subjects with quadratic teeth, a flat gingival margin, a broad zone of keratinized tissue, large interproximal contact located more apically and a thick alveolar bone, and “Thick-scalloped biotype” for subjects with thick gingiva with slender teeth, a narrow zone of keratinized tissue and a high gingival scallop (6-7).

Variations in bone and gingival architecture may lead to different tissue responses. In general, patients with a thin biotype are considered to have a higher risk of aesthetic complications after surgical or restorative treatments (1,2,7-9). On the other hand, thicker biotypes can originate gingival regrowth and poorer outcomes (3).

Different methods have been described to assess the gingival biotype, but most of them rely exclusively on the judgment of the examiner. Visual assessment taking into consideration the shape of the teeth, the aspect of the attached gingiva, and the position of the contact points between teeth is probably the simplest and commonly used method. All these clinical features are indirectly used to assess gingival thickness. Kan *et al*. (10) described a method involving the placement of a periodontal probe inside the gingival sulcus to assess its transparency. Depending on whether the contour of the probe could be seen or not, the biotype was classified as “thin” or “thick”, respectively (9,11). Finally, transgingival probing allows direct measurement of gingival thickness. This invasive technique provides more accurate assessment, but distortion may also occur due to inadequate needle directioning (i.e., not perpendicular).

Other authors have used different technologies to identify the gingival biotype (7-13). Müller *et al*. (7) suggested the use of ultrasound to assess the thickness of the masticatory mucosa. This technique was seen to be reliable, but not accurate for small changes (12). Radiographic three-dimensional (3D) techniques, specifically cone-beam computed tomography (CBCT), have also been used for tissue measurement (14). This method allows millimetric measurements, though it has considerable cost and exposes patients to radiation.

Although many authors insist on the importance of evaluating the gingival biotype in order to detect cases at an increased risk of esthetic failure after dental implant therapy, the data on assessment methods are scarce. Thus, the objectives of this study were to determine the reliability of three different gingival biotype assessment methods (direct visual analysis, placing a probe inside the gingival sulcus, and transgingival probing); identify the most commonly used method; and assess the importance of such diagnostic tools as rated by dental professionals based on the their own experience.

## Material and Methods

- Study design

A cross-sectional study was conducted based on an e-mail survey using specific software (SurveyMonkey, Palo Alto, USA), which was sent to all members of the Catalonia Dental Association (Col·legi Oficial d´Odontòlegs i Estomatòlegs de Catalunya – COEC, Barcelona, Spain). All participants were requested to assess the gingival biotype of 5 patients through 5 frontal view images (visual assessment), 5 sulcus area images (using the periodontal probe) and 5 images with the information of the gingival thickness in millimeters (a total of 15 images were shown to each participant). The participants were unaware that the 3 images were taken from the same patient using different methods. Clinical pictures were shown in a randomized order and participants were requested to classify all 15 images in “thick”, “thin” or “not able to be classified”. The concordance of the three methods was analyzed (frontal view, sulcus view and thickness in mm) using the answers for each case. Participants were invited to answer the questionnaire voluntarily and anonymously.

- Study questionnaire

**Figure 1:**
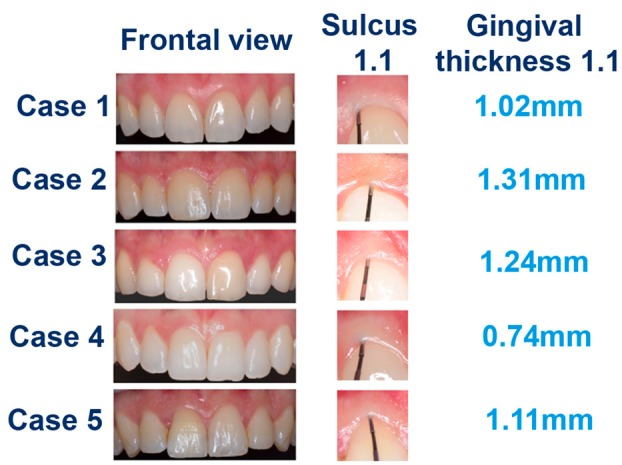
Photos and gingival thickness (in millimetres) of the 5 cases.

The questionnaire comprised 21 questions. The first 6 questions addressed the general characteristics of the dentists (professional experience, specialty, placement of dental implants) and gingival biotype evaluation. The remaining items were related to the analysis of the 5 cases and their classification according to gingival biotype (Fig. 1).

The participants could not change their answers once the survey was completed. Incomplete surveys were excluded from the analysis.

- Description of the cases

Five cases with different gingival thicknesses were selected. All of them had natural dentition in the anterior sector of the maxilla. Patients were non-smokers, were periodontally healthy, and had no restorations or crowns that could affect the gingival margin. Periodontal health was defined as no probing attachment loss, probing pocket depth of <3 mm, bleeding on probing <10% and no radiological bone loss (15). Three different assessment systems were applied to each case, and digital images were obtained with standardized parameters (1/160 seconds, F32, ISO 200, auto white balance) and using the same camera (Nikon® D5300, objective: AF-S DX micro Nikkor 85mm F/3.5G ED VR; flash: Macro Ring TTL Wireless Flash Slave Unit S1 S2; Tokyo, Japan). Photos were taken under artificial light.

The gingival thickness assessment methods were:

1. Visual assessment (VA) (8)

For visual assessment, lip spacers were used to allow correct visualization of the dental crowns, attached gingiva and mucosa. The photographic record included the upper anterior teeth (maxillary canines and incisors).

2. Assessment with a periodontal probe (APP) (10)

A CP-12 Hu Friedy® periodontal probe (Hu Friedy, Chicago, USA) was used in all cases. The probe was inserted in the middle area of the gingival sulcus of the central incisor. The photographic record was centered on the gingival margin of the tooth. The examiner could not observe the entire dental crown, gingiva and mucosa of the neighboring teeth.

3. Direct measurement (DM)

Endodontic no. 10 K-files with a rubber stopper were used for the direct measurements. The file was inserted in the middle area of the upper central incisor at a distance of 1.5 mm from the gingival margin. Local anesthetic was previously administered over this area (20% benzocaine gel; Hurricaine® gel, Clariben, Madrid, Spain). A rubber stopper was placed in contact with the gum. A CP-12 Hu Friedy® periodontal probe (Hu Friedy, Chicago, USA) was used as calibrator. The removed file was placed parallel to the periodontal probe and a photograph was taken for subsequent measurement using imaging software (Image J 1.46r; National Institutes of Health (NIH), Bethesda, USA).

The gingival thicknesses of the 5 cases were 0.74 mm, 1.02 mm, 1.11 mm, 1.24 mm, and 1.31 mm (Fig. 1). Since there is no gold standard technique to evaluate this variable, none of the employed methods was considered as a reference measurement.

- Statistical analysis

A descriptive analysis was made. Cohen’s kappa coefficient was used to measure agreement among the different assessment systems. The scale proposed by Altman (16) was used to interpret the kappa coefficient value. Kappa values were classified as Poor (0-0.20), Weak (0.21-0.40), Moderate (0.41-0.60), Good (0.61-0.80) or Very good (0.81-1.00). Comparisons of proportions were made using the chi-squared test. Statistical significance was considered for *p* < 0.05.

## Results

A total of 116 professionals participated in the study. Twelve surveys were excluded due to incomplete data. A total of 104 surveys were thus finally analyzed.

The main characteristics of the participants are shown in Table 1. The perceived diagnostic importance of gingival biotype differed (*p* < 0.05) according to the specialty of the respondents, their years of experience, and whether they placed dental implants or not. Gingival biotype was considered to be an important or very important variable to 78% of the specialists and 40% of the general dentists, and by 81% of those who placed dental implants versus 50% of those who did not. Experienced professionals also considered this variable has being relevant (less than 5 years of experience: 54%; 5-10 years: 71%;> 10 years of experience: 78%).

The most commonly used method to classify the gingival biotype was the visual assessment of the teeth and gingival morphology (62.5%), followed by the use of a periodontal probe (49%). Only 9.6% of the participants measured gingival thickness directly. A minority also used other methods to identify the biotype, such as CBCT (1%) and measurement of the amount of keratinized tissue (1%).

**Table 1:**
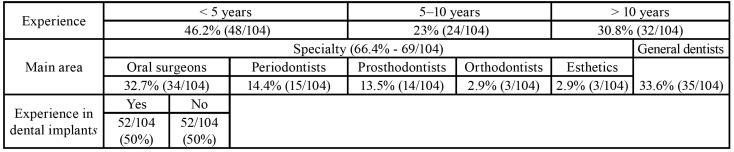
Principal characteristics of the participants.

**Table 2:**
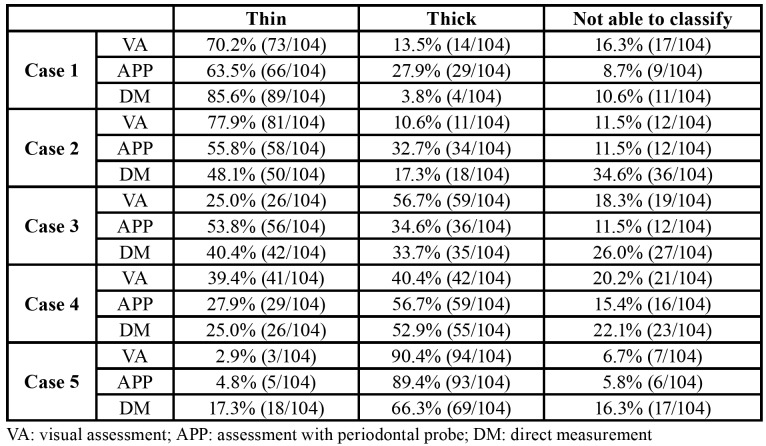
Responses with the three evaluation systems in each case.

- Gingival biotype assessment

The participant answers are summarized in Table 2.

The three assessment methods showed a maximum kappa coefficient of 0.278 (weak) between visual assessment and assessment with a periodontal probe; 0.184 (poor) between visual assessment and direct measurement; and 0.108 (poor) between direct measurement and assessment with a periodontal probe. The minimum and maximum kappa values among assessment methods were calculated using the answers of the 5 cases together (Table 3).

**Table 3:**
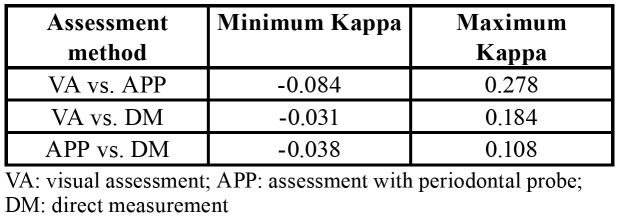
Minimum and maximum Cohen kappa values between systems.

Percentage agreement among all the systems was greater in the cases characterized by thinner and thicker gingival thickness, with no significant differences according to the experience of the professional (Table 4).

**Table 4:**
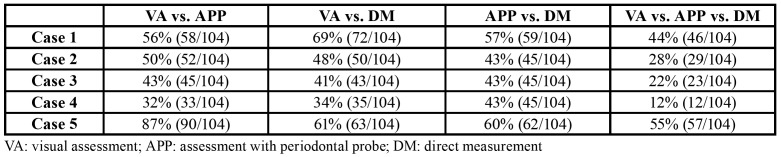
Percentage of concordant answers between systems.

## Discussion

The results obtained in our study show that the three assessment methods commonly used to determine the gingival biotype presented poor to weak concordance, with a maximum kappa value of 0.278.

The strongest agreement was found between visual assessment and assessment with a periodontal probe – these being the most commonly used methods for assessing the gingival biotype among the participants. The real thickness (measured in mm) obtained through direct measurement showed lower agreement when compared with the other two methods. These results differ from those obtained by Kan *et al*. (17), who compared the same systems and found higher agreement between the periodontal probe and direct measurement. On the other hand, visual assessment showed significant differences versus the other methods. Kan *et al*. (17) established a threshold for classifying the gingival biotype (thick biotype > 1 mm and thin biotype < 1 mm), and this might partially explain the differences with respect to our own data. In the present study, no information was given to the participants to avoid influencing the answers. Furthermore, there is no universally accepted limit within the dental community for this variable, so providing values could lead to a classification bias.

As expected, the more extreme cases showed the highest number of concordant responses, without differences taking into consideration the experience of the professional. However, great variability was found, particularly in the more intermediate cases, with an important lack of agreement among the participants. Fischer *et al*. (11) reported similar findings, with significant differences in gingival thickness in the most extreme groups. Thus, the current gingival biotype classification clearly needs revision.

Visual assessment is generally the most widely used method among the professionals. It is a simple, rapid and straightforward system allowing the evaluation of several parameters of the teeth and surrounding tissues. However, it does not seem to be reliable in classifying the gingival biotype (13,17). Indeed, in the present report, only one case (thick biotype) was identified correctly by most respondents. Thin-scallop biotypes are considered to be a risk factor for esthetic complications after dental implant placement. However, these biotypes are poorly classified in more than half of all cases. Fisher *et al*. (11) studied the relationship between gingival thickness and gingival biotypes, and excluded participants with different gingival biotypes in the two upper central incisors - indicating that gingival thickness is individualized to each area and cannot be assessed by jointly observing the macroscopic (gross) dental and gingival characteristics of the anterior maxilla.

Almost 50% of the respondents reported the use of a periodontal probe. Kan *et al*. (17) introduced this system and found it to be an objective evaluation tool, especially when compared to direct measurement. Both Kan *et al*. (17) and De Rouck *et al*. (6) confirmed the accuracy and reproducibility of this technique. However, the present study recorded poor agreement between the periodontal probe and direct measurement. It should be noted that these results might be influenced by problems related to the analysis of gingival thickness through direct measurement rather than to observation of the transparency of the periodontal probe. Again, the thick biotype case showed the best agreement. These results are consistent with those of other authors (13,14) who concluded that the periodontal probe is of limited diagnostic value for this parameter.

Direct measurement of the gingiva was rarely used by the clinicians. It exhibited greater response variability, possibly due to the absence of a standard gingival thickness threshold in mm. Indeed, this fact justifies the poor agreement of this method with the other two techniques. Biotype categorization according to gingival thickness differs widely in the literature, with thresholds ranging from 1 mm to 2 mm (16-18). However, Fischer *et al*. (11) found maximal thickness of the gingiva to be 0.92 mm; thus, according to the criteria described in the above-mentioned studies, all their cases would be considered as a thin biotype. Once again, this outcome shows the need to establish a commonly accepted classification for gingival biotype. In the present sample, the cases had a thickness ranging from 0.74mm to 1.31mm. Since extreme cases (for example, gingival thickness of more than 2mm) are quite rare and generate less classification doubts, the authors of the present study decided to select cases with more subtle differences in order to increase the clinical applicability of the results.

In the present sample, more than 85% of the respondents clearly classified the thin biotype when the thickness was < 1 mm. However, variability increased when the thickness was greater.

More experienced professionals, especially those who place dental implants, tend to define the gingival biotype as an important variable that should be considered in the diagnosis and in treatment planning.

The different measurements of the cases were shown to the clinicians though images. This might be considered a limitation, since a three-dimensional observation could improve the diagnosis. On the other hand, this methodological approach allowed for a large study sample.

The present study shows that the methods currently used to classify gingival biotypes clearly lack inter-examiner reproducibility. This is an extremely important observation, since gingival biotype is directly correlated to the esthetic outcome of treatment (1,7,9). Thus, further studies are needed to define more accurate and reproducible methods for assessing the gingival biotype.

In conclusion, the most frequently used assessment methods for classifying the gingival biotype are not reliable and lack inter-examiner reproducibility. There is a clear need to define new diagnostic criteria and to develop more reliable assessment systems.
